# Conference Reports: INTERNATIONAL CONFERENCE ON NARROW-GAP SEMICONDUCTORS AND RELATED MATERIALS Gaithersburg, MD June 12–15, 1989

**DOI:** 10.6028/jres.095.037

**Published:** 1990

**Authors:** D. G. Seiler, C. L. Littler

**Affiliations:** Semiconductor Electronics Division, National Institute of Standards and Technology, Gaithersburg, MD 20899; Department of Physics, University of North Texas, Denton, TX 76203

## 1. Introduction

The Semiconductor Electronics Division at the National Institute of Standards and Technology (NIST) hosted an International Conference on Narrow-Gap Semiconductors and Related Materials in Gaithersburg, MD on June 12–15, 1989. A brief background on narrow-gap semiconductors (NGSs) is given in this paper, along with an overview of the conference itself. The major section of this report is devoted to highlights from each of the invited papers in order to put this field of semiconductor research and technology in perspective. The Conference Proceedings has been published as a special issue of Semiconductor Science and Technology (Institute of Physics Publishing, Bristol, March 1990, Volume 5).

### 1.1 Background on Narrow-Gap Semiconductors

Semiconductors are an important class of materials that not only are of scientific interest, but also have a wide variety of useful, technological applications in our modern-day world. Semiconducting phenomena have been detected in several basic forms of matter including crystals, glassy and amorphous solids, liquids, and even organic materials. A definition of a semiconductor that includes all of these materials would be somewhat too general to be useful. A narrower but more often-used definition involves the concept of a crystal which is composed of a periodic array of atoms. Here, a semiconductor is defined as a solid, crystalline material with predominantly covalent bonding and electrical conductivity that is intermediate between that of a metal and that of an insulator. The conductivity is usually strongly temperature dependent over some range of temperatures.

What are NGSs? The energy band theory of solids allows this classification of a semiconductor material in terms of the size of its fundamental energy gap, *E*_g_, the separation between the energy of the lowest conduction band and the highest valence band. W. Zawadzki [1] has defined an NGS as a material in which the energy of an occupied electron or hole state, as measured from the corresponding band extremum, can become comparable to the energy gap, *E*_g_. According to this definition, any semiconductor can exhibit “narrow gap” properties under appropriate conditions (e.g., if electrons are optically excited sufficiently high above the band edge). However, a more traditional definition would identify NGSs as solids having energy gaps of less than 0.5 eV [2]. With this last definition, it is clear that NGSs are found among a broad spectrum of elements, compounds, alloys, and artificially structured materials. Table 1 gives some representative examples of NGSs. Improvements in crystal growing techniques have led to the production of many different types of alloys whose energy gaps can be adjusted by varying the composition. In particular, the emergence of molecular-beam epitaxy (MBE) technology has opened up the new field of band structure (or band gap) engineering. The MBE process refers to a technique in which several atomic (or molecular) beams impinge on a heated substrate material under ultrahigh vacuum conditions. With the growth of new artificial semiconductor structures by MBE technology, the transport, optical, and other properties of the electrons and holes can be altered continuously and independently, leading to interesting new physics and new classes of semiconductor devices. Single crystalline layers are grown in registry with the substrate, permitting highly controlled epitaxial growth. Numerous specific examples of MBE-grown materials were presented at the conference.

NGSs have long been recognized for their special characteristics that give rise not only to interesting physical effects for basic studies but also to useful technological applications. In 1957, E. O. Kane [3] showed how to describe the band structure of NGSs by using the *k* · *p* method. This approach uses perturbation theory in conjunction with crystal symmetry requirements to investigate the wave functions and the form of the energy bands in the vicinity of high-symmetry points in *k*-space. The corresponding band structure can then be determined by obtaining a limited number of parameters experimentally such as energy gaps, electron and hole effective masses. This method is of considerable importance because it has a firm theoretical foundation and has been found to describe most of the semiconductor properties related to band structure. The small energy gap characteristic of an NGS usually means that the electron effective mass m* is also small. Values of m* less than 0.001 *m*_0_ have been observed, where *m*_0_ is the free electron mass. Small values of m* lead to large carrier mobilities and many other interesting physical properties such as high carrier saturation velocities, strong nonlinear optical effects, strong magnetic quantum effects, etc. The properties of an NGS are also more sensitive to external influences such as temperature, magnetic field, electric field, or strain, than are the larger band gap semiconductors. Some of these NGSs are used extensively as infrared detectors, particularly those that are sensitive to the regions of the electromagnetic spectrum corresponding to the atmospheric windows at 3 to 5 µm and 8 to 14 µm.

### 1.2 Applications of Narrow-Gap Semiconductors

Two major applications of narrow-gap materials are infrared detectors for passive imaging and diode lasers for high-resolution spectroscopy. Other applications include Hall probes for measuring magnetic fields and thermoelectric devices for power generation and cooling purposes. NGSs are also used by radio astronomers as sensitive, reliable, and comparatively fast submillimeter detectors. We briefly review the two major applications.

#### 1.2.1 Infrared Detectors

The electromagnetic radiation spectrum lying between the visible and microwave frequencies is generally defined as the infrared (IR) region. Its importance arises from the fact that every material object emits, absorbs, transmits, and reflects infrared radiation in a characteristic manner. From a study of the intensity and wavelength distribution of the radiation which has arisen from or interacted with an object, information concerning the object may be obtained. This information can be used to distinguish a body from its surroundings or to identify an unknown material. Military applications in IR technology are important for several reasons: (1) most targets of interest (vehicles, troops, etc.) differ from the surrounding terrain either in temperature or emissivity or both and thus can be seen readily by IR equipment; (2) IR systems can utilize the radiation emitted by the targets they seek (in a passive manner such that the detection system does not disclose its presence the way a radar system does); (3) IR is capable of revealing greater detail than radio or radar waves because of its shorter wavelengths. The key component of IR systems is the detector itself.

IR detectors are devices that convert electromagnetic radiation to conductive electric signals which can then be processed to obtain the information inherent in the temporal and spatial variations of the radiation. Detectors may be classified as either thermal or photon detectors. The absorption of radiation in thermal detectors such as bolometers or thermopiles produces an increase in temperature which then can be measured. In photon detectors, electrons or holes, or both, are created by the absorption process, thus producing carrier concentrations and conductivities greater than the thermal equilibrium values. Important photon detectors are based upon characteristics or effects such as photoconductivity, photovoltaic effect, photoelectro-magnetic effect, and charge-transfer effects (i.e., metal-insulator-semiconductor (MIS) structures). Photon detectors are greatly superior to thermal detectors in terms of speed of response and sensitivity.

IR detectors are used extensively in military night-vision systems and remote temperature sensing. Interest also exists in heat sensing for home and industrial energy loss, medical thermography (e.g., breast cancer detection), for astronomical research, and in spectrophotometers.

#### 1.2.2 Diode Lasers

Lead-salt (i.e., PbS, PbSe, and PbTe) diode lasers have proven to be extremely valuable tools in high- resolution molecular spectroscopy with related applications to air pollution monitoring. A direct energy gap is a requirement for efficient radiative recombination and stimulated emission. The emission occurs in the infrared region at photon energies close to *E*_g_, and the laser wavelength can be adjusted with high precision and often over wide ranges by varying the temperature, magnetic field, or pressure in order to change *E*_g_. Diode lasers of PbS_1−_*_x_*Se*_x_*, Pbi_1−_*_x_*Sn*_x_*Te, or Pb_1−_*_x_*Sn*_x_*Se are typically used as sources of coherent infrared radiation with wavelengths between about 4 to 30 µm. Their spectral output has an extremely narrow linewidth which is particularly attractive for high-resolution spectroscopy. Work continues today on increasing their operating temperatures, improving their output characteristics, and increasing the device operating lifetime.

## 2. Previous Narrow-Gap Conferences, Conference Organization, Sponsors, and Attendance

Conferences are important not only for reviewing and summarizing past research, but also for providing an overview of the current state of the art. In addition, a conference can be an important stimulus to new progress in the field. As can be seen from table 2, no conference devoted entirely to NGSs has been held since the one in Linz, Austria, in 1981. The date, place, sponsors, and published proceedings are listed there. New materials and new phenomena have subsequently arisen and, consequently, new perspectives in the field of NGSs were obtained by holding this conference. Important papers were given at the Gaithersburg Conference in 1989, covering many new materials and phenomena. The considerable progress made since the previous meeting in 1981 was reviewed, clarified, and elucidated in numerous invited talks.

The Conference Chair was David G. Seiler, Group Leader of the Materials Technology Group at the National Institute of Standards and Technology. Mike Kinch of Texas Instruments was the Program Committee Chair and Chris L. Littler the Conference Treasurer. Sponsors of the Conference were the U.S. Air Force Office of Scientific Research, American Physical Society, National Institute of Standards and Technology, National Science Foundation, U.S. Office of Naval Research, Texas Instruments, and University of North Texas. The session chairs came from well-known government, industrial, or university laboratories.

A tour through several major semiconductor laboratories of NIST’s Semiconductor Electronics Division (SED) was hosted by David G. Seiler and Frank Oettinger, Division Chief. The SED conducts experimental and theoretical research on semiconductor materials, devices, and integrated circuits. The tour concentrated on visits to labs and on presentations on deep-level metrology, MBE work, intelligent test structure metrology, and ellipsometry.

One hundred fifty-nine attendees from 14 different countries participated in the conference sessions. Seventy-two contributed and 14 invited talks were given on a wide range of bulk, film, and artificially structured materials: II–VI, III–V, IV–VI compounds and various alloy semiconductors. Topics covered ranged from IR detector device physics to the growth and characterization of artificially structured materials, as well as a review of high-*T*_c_ superconductors as IR detectors. As judged by the attendance at the conference, the quality of the talks given, the papers submitted for publication, and the oral and written comments from the attendees, the conference was a great success.

## 3. Highlights from Invited Papers

In this section we present some highlights selected from the invited papers given at the conference. No attempt is made to be comprehensive in these short reviews. We hope that these highlights will be interesting enough that many readers will wish to examine the complete Conference Proceedings published in Semiconductor Science and Technology, March 1990.

*Bill Paul* (Harvard University) opened the NGS Conference with some stimulating remarks and perspectives on this class of materials. He pointed out that attention to the details of the materials preparation and chemistry is essential to a determination of the correct physical properties. Furthermore, good materials preparation has been the springboard for most of the new advances in the NGS field. A review of studies on gray tin (*α*-Sn) was given (*α*-Sn was the progenitor of the whole class of zero-gap or negative-gap materials). The advent of MBE and of the production of thermodynamically metastable films may allow alloys such as Ge-Sn to be grown with direct gaps varying from about 0.5 eV to zero over a certain range of composition. Many superlattices (SLs) of the types Sn/InSb, Sn/CdTe, Sn*_x_*Ge_1−_*_x_*/Ge, Sn*_x_*Si_−_*_x_*/Si or Sn/Hg*_x_*Cd_1−_*_x_* Te remain to be made and studied. He concluded that the maturing fabrication techniques for quantum wires and dots (made possible by different kinds of lithography) have already resulted in new physical phenomena and new devices and will continue to do so. One can say confidently that there are unimagined phenomena beyond the horizon, ensuring the need for meetings such as this one well into the next century.

**Figure 1:**
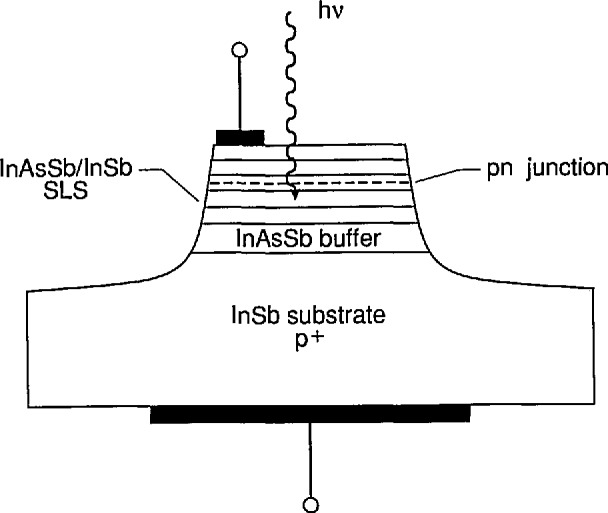
Schematic illustration of the InAsSb strained-layer-superlattice photodiodes fabricated at Sandia National Labs (see Conference paper of G. C. Osbourn, fig. 3). The SL is grown on a *p*-type InSb substrate with an intervening InAsSb graded buffer layer. As shown, the *pn* junction occurs within the SL structure. These SL materials can be used as direct replacements for bulk materials in standard photovoltaic or photoconductive device structures.

*Gordon Osbourn* (Sandia National Laboratories) reviewed the multi-quantum well (MQW) and superlattice (SL) physics which influences device performance and described the status of the leading III–V approaches for long-wavelength detection. Modern growth techniques have made possible the preparation of high-quality, single-crystal layered structures, with layer thicknesses ranging from micrometers to nanometers. These structures may provide useful alternatives to the II–VI Hg_1−_*_x_*Cd*_x_*Te alloy system for long-wavelength detector applications. Detector technology based on III–Vs will benefit from superior bond strengths and material stability, well-behaved dopants, and high-quality III–V substrates. The MQW and SL heterostructures (HSs) have new and interesting energy band structures which depend on layer thicknesses, layer strains, and the bulk properties of the individual layers. Research has revealed a number of mechanisms for obtaining long-wavelength cutoffs in the following structures: (1) materials with “type II” band offsets (BOs) where transitions occur between hole states in one type of layer and electron states localized in the other type; (2) doping SL material with a series of back-to-back p-i-n junctions; (3) quantum-well materials with transitions occurring between the ground and first excited states in the conduction band, the energies of which are determined by the quantum size effect; and (4) strained-layer superlattice (SLS) structures with a type II BO. SLSs, such as those in the InAsSb system, are grown from lattice-mismatched alloy layers with layer thicknesses thin enough to allow complete elastic strain accommodation of the mismatch. A schematic illustration of an InAsSb SLS photovoltaic detector fabricated at Sandia is shown in figure 1. These SLS structures require minority carrier collection across the SLS layers at low temperatures for good quantum efficiencies. The carriers are collected directly from the depletion region or by diffusion from the neutral regions to the depletion region edge. Other structures reviewed were AlGaAs/GaAs, InAs/GaSb SLs, InAs/InGaSb SLSs, and InSb nipi SLs. All materials systems will require significant improvements in their detectivity values before competitive detector technologies can arise.

**Figure 2:**
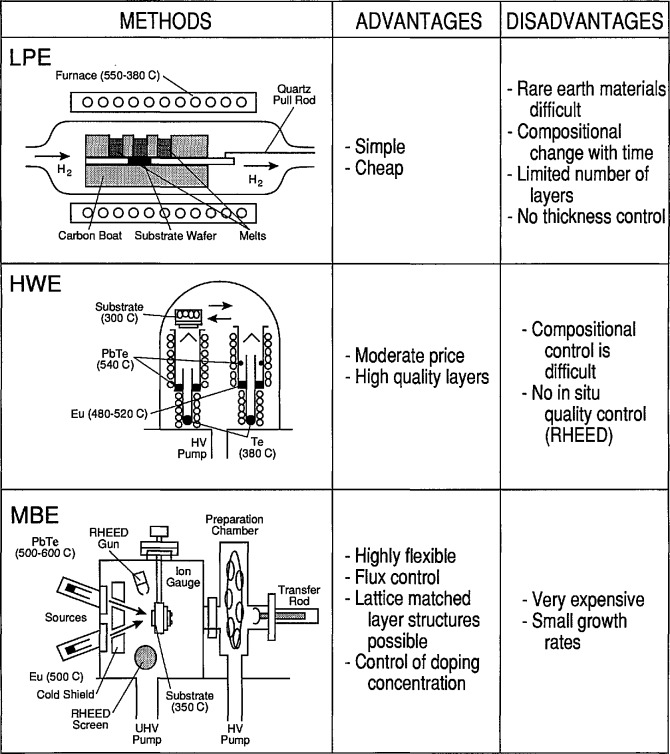
Comparison of three epitaxial growth techniques: liquid phase epitaxy (LPE), hot wall epitaxy (HWE), and molecular beam epitaxy (MBE) (see Conference paper by H. Preier, fig. 3). In the LPE method, growth occurs by controlled lowering of the melt temperature. No MQW and single QW structures can be produced by the LPE method. The HWE method is a vapor-phase epitaxy technique where substrate and source form a quasi-enclosed system. Layer structures are grown by positioning the heated substrate on top of different source furnace arrangements. As shown in the diagram, PbTe layers can be grown on the right side and PbEuTe layers on the left side. The temperature of the Eu furnace controls the Eu content, that of the Te furnace the doping concentration. Multiple-layer structures can be produced by switching the substrate back and forth. Using the MBE method, layers of various compositions can be deposited by properly combining molecular beams from different source ovens.

**Figure 3:**
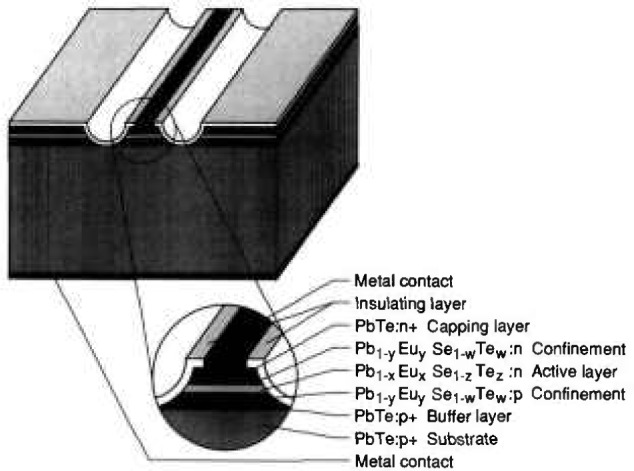
Schematic view of a typical device structure of an MBE grown PbEuSeTe/PbTe double heterostructure laser grown at Laser Analytics (see Conference paper by H. Preier, fig. 3). Note that metal contacts have to be applied only to heavily doped PbTe and that the current is restricted to a narrow stripe region by mesa etching.

*Horst Preier* (Johannes Kepler Universität) reviewed the state of the art and the development trends of lead salt lasers. They are the key component in diode laser spectrometers. Scientific research in fields like high-resolution spectroscopy (of molecules, ions, and radicals), sub-Doppler spectroscopy in molecular beams, and heterodyne and acousto-optic spectroscopy relies on these diodes. Diode laser spectrometers are also used (1) in the automotive industry for time-resolved car exhaust-gas studies and for trace gas analyses, (2) for smoke stack monitoring of power plants for the detection of trace gases like NH_3_ in the exhaust stream of catalytic converters, and (3) in medical diagnostics for isotope-specific analyses of exhaled breath after being treated with food or drugs labeled with stable isotopes (giving information about metabolism). The entire wavelength range from 3 to 30 µm can be covered by using ternary and quaternary compounds like PbSnTe, PbSSe, and PbEuSeTe. These lasers can be easily tuned by temperature or current and have narrow linewidths of ≈10^−4^ cm^−1^. Only 12 institutions worldwide have been or remain involved with lead salt laser activities. The lasers have been produced by liquid phase epitaxy (LPE), hot wall epitaxy (HWE), and MBE. The advantages and disadvantages of each of these methods are summarized in figure 2. The most sophisticated and expensive technique is the MBE method. Shown in figure 3 is a typical device grown by MBE technology at Laser Analytics. The compositional control is very good, allowing exact lattice matching, and close control of the doping concentration. In-situ monitoring of the crystalline quality of the growing layers can be accomplished by RHEED analysis. Accurate thicknesses are produced by low growth rates, but correspondingly long manufacturing times are then needed to produce the laser structure. The dominant development goal has been to increase the operating temperature to the point that only inexpensive cooling equipment is needed. Improvements in the understanding of the basic material properties of these ternary and quaternary compounds are needed to establish better theoretical models for the threshold currents.

*C. Tom Elliott* (Royal Signals and Radar Establishment) reviewed research work on non-equilibrium modes of operation of semiconductor devices using the phenomena of minority-carrier extraction and minority-carrier exclusion to reduce carrier densities in narrow-gap HgCdTe alloys to near “extrinsic” values at temperatures where the materials are normally intrinsic. By this means Auger noise is suppressed; it would ordinarily be a severe limiting factor for IR detectors operating at near-ambient temperatures. Experimental work on excluding-contact photoconductors (one type of non-equilibrium mode device used to study the Auger suppression) showed signal-to-noise improvements at high modulation frequencies which are consistent with the theoretically predicted Auger suppression mechanism. Bipolar transistor action, observed at temperatures where the material is near intrinsic, indicates the potential of NGSs for devices other than IR detectors when operated in a non-equilibrium mode at ambient operating temperatures. In ordinary intrinsic semiconductor IR detectors, thermal processes compete with optical processes in the generation of free carriers and the purpose of cooling is to reduce the thermal generation and its associated fluctuations and noise. Currently, high-sensitivity detectors for the 8- to 12-µm region are typically operated at 80 K, while those for the 3- to 5-µm region are operated around 200 K. These cooling requirements add considerably to the cost, size, and inconvenience of IR systems. The experimental and theoretical work on steady-state non-equilibrium operation described here may open up possibilities for more widespread use of NGSs.

*Peter Wolff* (Massachusetts Institute of Technology) described free-carrier-induced optical nonlinearities of NGSs. There are three electronic mechanisms that give rise to large optical nonlinearities in semiconductors: nonlinear free-carrier dynamics, band filling, and exciton resonances. There are a variety of semiconductor nonlinear optic devices, e.g., bistable phase-conjugate elements, tunable filters, and power limiters. Exciton resonances in AlGaAs/GaAs SLs have large, room-temperature optical nonlinearities and are currently the most promising for optical signal-processing devices needed for all optical computers. NGSs generally have large nonlinear optic coefficients. Comprehensive theoretical and experimental details of free-carrier optical nonlinearities were presented along with nonlinearities due to interband transitions in zero-gap materials and carrier-scattering effects.

*Jerry Meyer* (Naval Research Laboratory) reviewed the present understanding of band-edge and free-carrier properties in Hg-based SLs such as HgTe/CdTe. This field of study has blossomed since the first successful MBE growth of these SLs in 1982. Novel phenomena have been discovered which are distinct from anything observable in either Hg-based alloys or in wide-gap SLs. He emphasized the relation between the distinctive aspects of the SL band structures obtained theoretically and corresponding features in magneto-transport, magneto-optical, and optical data. Theoretically, there is a high sensitivity of the free carrier properties to the magnitudes of the valence band offset. For example, the identity of the dominant hole band depends on whether the valence band offset is large or small. He argued that most experimental results are qualitatively consistent with a large offset. However, there is a strong need for more detailed quantitative theories to explain the numerous unusual features of the band structure, such as mass broadening (carriers coexisting with a wide range of in-plane masses).

**Figure 4:**
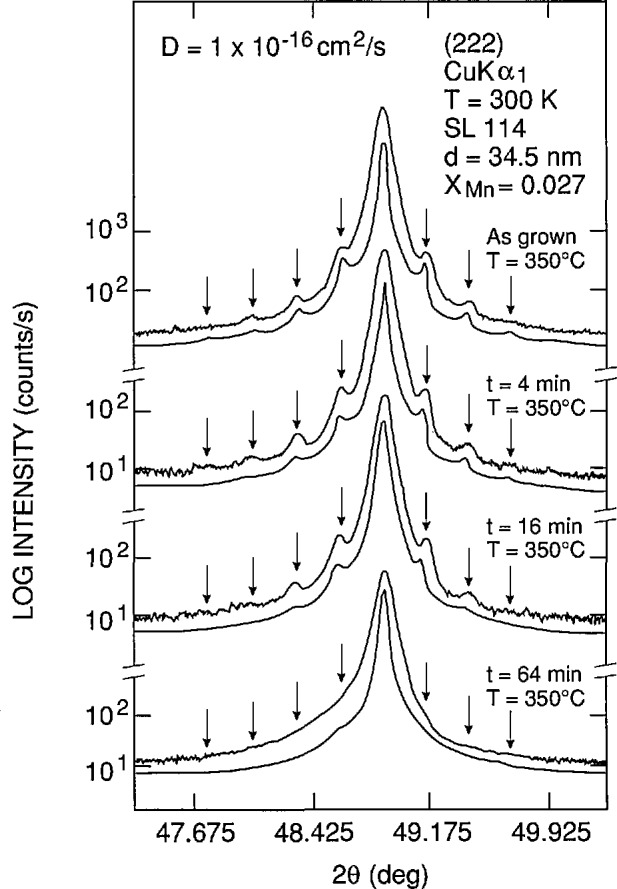
Example of the use of x-ray diffraction techniques to characterize MBE-grown SL structures. X-ray diffraction Bragg intensities versus angle of incidence for a Pb_1−_*_x_*Mn_s_Te structure (x = 0.027, 20 periods t_pbTe_ = 4.5 nm, t_PbMnTe_ = 30 nm) (see Conference paper by G. Bauer, fig. 4). The experimental data compare favorably with the calculated intensities shown below each data curve. The influence of the annealing time on the satellite peaks can be directly seen as discussed in the text.

**Figure 5:**
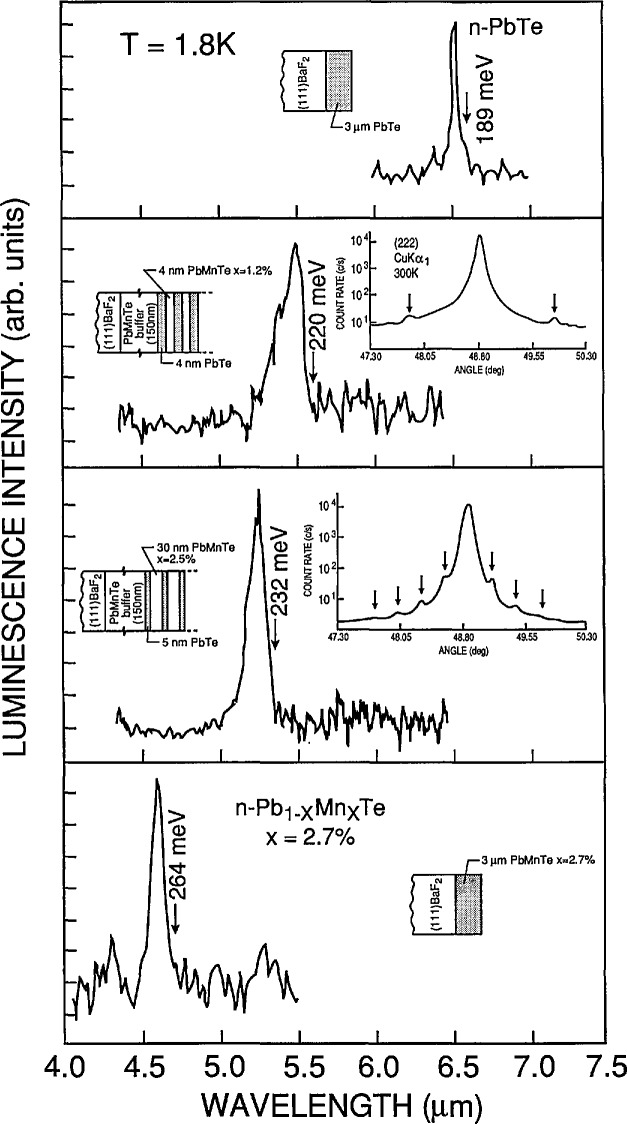
Characterization of various MBE-grown structures by photoluminescence techniques for PbTe and Pb_1−_*_x_*Mn*_x_*Te films and two QW structures. The geometry of each structure is shown along with x-ray intensity versus angle data for the two QWs. As discussed in the text, these data give evidence for the quantum confinement effects that are present in certain structures.

*Günther Bauer* (Montanuniversität Leoben) reviewed some of the interesting experimental investigations that have been carried out on IV–VI compound QW and SL structures. QW structures of these materials were first grown in 1980. The main application of these materials is for mid-IR lasers with relatively high operating temperatures as compared to those made from III–V materials. Many II–VI and IV–VI NGSs have large diffusion coefficients, and experiments that can give information on the diffusion constant *D* are important to carry out. Figure 4 shows the results of x-ray diffraction experiments on a PbTe/Pb_1−_*_x_*Mn*_x_*Te SL sample with *x* =0.027 consisting of 20 periods with thicknesses *t*_PbTe_=4.5 nm and *t*_pbMnTe_=30 nm. The curves for Bragg intensity versus angle exhibit satellite peaks which can be analyzed to give information on the period (34.5 nm) as well as on inter-diffusion (e.g., values for *D*). With increasing annealing times, more and more satellite peaks are smeared out; this directly shows the increase of *D.* The calculated scattered x-ray intensities are shown below each experimental curve. These kinds of experiments imply that close to the substrate, inter-diffusion will be more severe than close to the surface, since layers in the vicinity of the substrate have been at the growth temperature for a longer period of time. Thus the electronic properties will be very different for wells close to the substrate from those close to the surface. Another interesting set of experiments showing quantum confinement effects in PbMnTe/PbTe structures is seen in figure 5. A Nd-YAG laser was used to excite luminescence in PbTe and PbMnTe films and in two QW structures. Since MnTe has a greater band gap than PbTe, the *x* =2.7% alloy has a greater gap than PbTe, and its photoluminescence peak appears at lower wavelengths or higher energies. For the sample with a period of 8 nm, a real SL is formed with finite dispersion along *k*_z_, whereas for the sample with 30-nm-thick PbMnTe barriers, the carriers are confined in the PbTe wells. BOs are important parameters to understand for the artificially structured materials, and Bauer points out that electron-beam-induced current techniques showed that the valence band offsets in both PbEuSeTe and PbEuSe structures depend drastically upon temperature. Finally, PbTe *nipi* structures have been shown to have large detectivity values (≃10^11^ cm Hz^1/2^ W^−1^), close to the background limit at 77 K.

**Figure 6:**
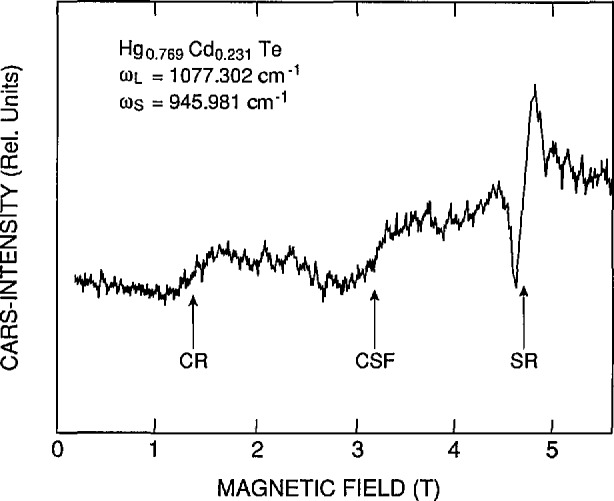
Example of using nonlinear optical techniques to characterize semiconductors. The CARS intensity of an *n*-type Hg_1−_*_x_*Cd*_x_*Te sample (*x* =0.231) is shown versus magnetic field (see Conference paper by H. Pascher, fig. 9). Various Ramanlike resonances appear at different magnetic fields and can be used to characterize the electronic structure of the material in a contactless manner. Cyclotron resonance (CR), combined spin flip (CSF), and spin resonance (SR) structures are shown.

**Figure 7:**
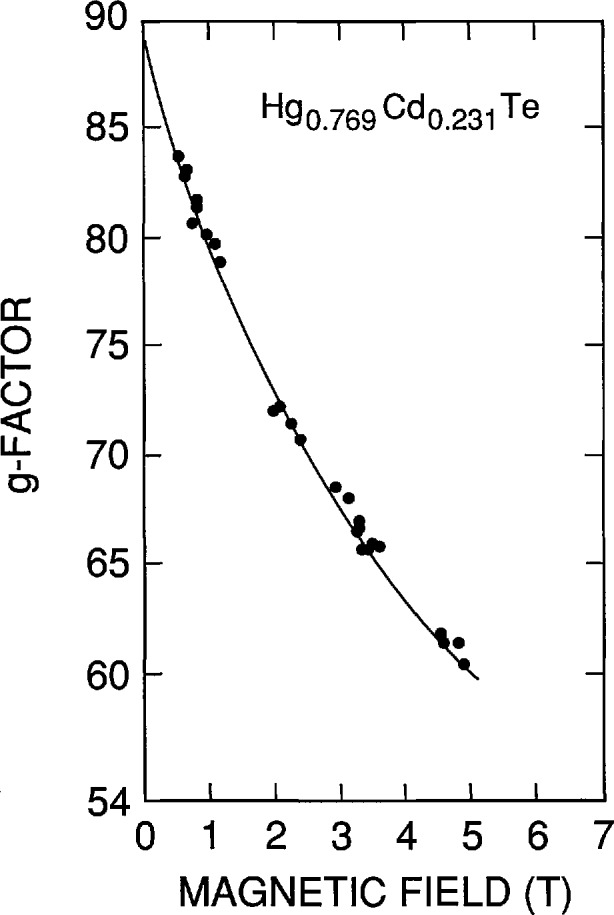
Effective *g*-factor of a Hg_1−_*_x_*Cd*_x_*Te sample (shown in fig. 6) versus magnetic field (see Conference paper by H. Pascher, fig. 10). The g-factors can be accurately calculated from the magnetic-field positions of the SR. The magnetic-field dependence of the g-factor is a direct consequence of the non-parabolic nature of the conduction band.

*H. Pascher* (Universität Bayreuth) reviewed the use of optical four-wave mixing Raman techniques for observing and studying magneto-optical intraband transitions in NGSs. Coherent anti-Stokes Raman spectroscopy (CARS) is one such technique that is well suited to characterize a wide variety of samples—bulk crystals, epitaxial films, QWs, and diluted magnetic semiconductors (DMSs). The strongest Raman-like resonances in a semiconductor exposed to a magnetic field are the spin-flip resonances from which the effective *g*-factors of electrons and holes can be deduced. Figure 6 shows an example of the CARS technique applied to an *n*-type Hg_1−_*_x_*Cd*_x_*Te bulk crystal (*x* =0.231) showing cyclotron (CR), combined spin flip (CSF), and spin resonances (SR). The effective *g*-factor of the sample can be extracted from the SR signals and is shown plotted versus magnetic field in figure 7. Thus, very precise band-structure information can be obtained by these techniques. In MQWs and SLs of the IV–VI compounds, the nonlinear susceptibilities are enhanced with respect to the corresponding bulk materials. Strong mixing signals can be observed even in samples which were only 2-µm thick. Studies of *g*-factors of a PbTe/Pb_1−_*_x_*Sn*_x_*Te MQW showed they were identical to those of a Pb_1−_*_x_*Sn*_x_*Te reference sample. This proved that the electrons were confined within the Pb_1−_*_x_*Sn*_x_*Te layers and thus the band alignment of the system is of type I.

*M. Dobrowolska* (University of Notre Dame) reviewed the phenomenon of spin resonance (SR) of conduction band electrons in NGSs. SR is defined as a magneto-optical transition in which the electron spin is flipped between two spin states belonging to the same Landau subband or to the same impurity state. It is important to investigate because (1) SR gives the most direct and accurate measurement of the *g*-factor, a parameter that provides detailed information about band structure and (2) SR is normally forbidden by electric-dipole selection rules, but perturbations (nonparabolicity, inversion asymmetry, and warping) relax these rules allowing insight into band-structure details. For a typical NGS at laboratory magnetic fields (*B* < 10 T), the SR transition corresponds energetically to the far-infrared (FIR) region accessible by FIR lasers. The review concentrated on InSb, but other materials such as HgSe, HgTe, Hg_1−_*_x_*Mn*_x_*Se were also briefly discussed.

**Figure 8:**
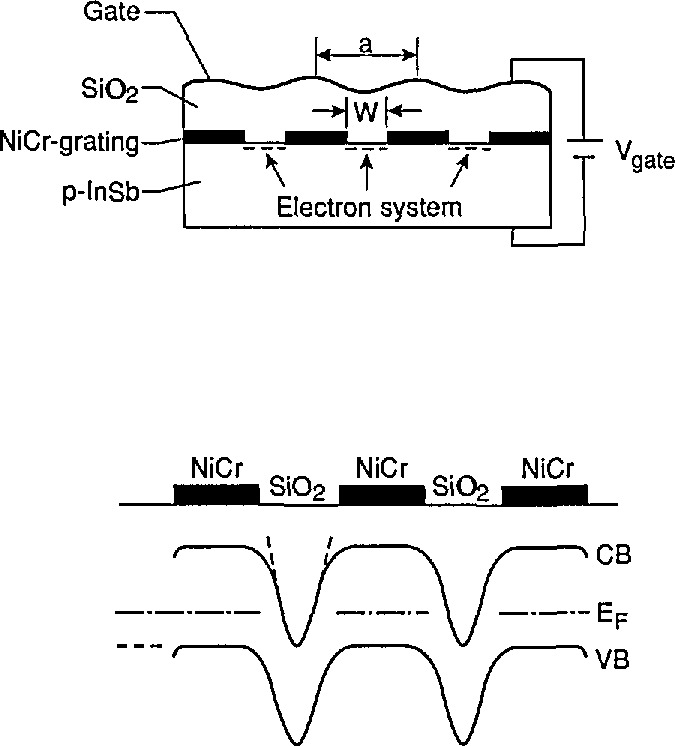
Schematic cross section of the microstructure field-effect device on InSb that is used for quantum wires and quantum dots (see Conference paper by U. Merkt and Ch. Sikorski, fig. 1). Basically it is an MOS capacitor. The lower half of the figure shows the corresponding band structure and Fermi level in the device.

*Ulrich Merkt* (Universität Hamburg) reported on his investigations of quantum wires and dots near the surface of InSb that contain quasi-one-dimensional and zero-dimensional electron systems. InSb and GaAs/Ga_1−_*_x_*Al*_x_*As heterojunctions are the first semiconductors in which electron systems can be studied in all four sets of dimensions from 3-D to 0-D. The 3-D system in InSb has been studied since 1952; the 2-D system began around 1970 with inversion layers (ILs) in metal-oxide-semiconductor (MOS) structures; and within the last 3 years 1-D and 0-D systems were created by laterally confining the ILs. Figure 8 presents the basic ideas of the quantum-wire and quantum-dot structures fabricated by Merkt. It is essentially an MOS capacitor configuration. NiCr is evaporated onto a *p*-InSb substrate using a photoresist mask. A Schottky contact is established at the NiCr/InSb interface and the Fermi energy *E*_F_ is pinned within the band gap. Mobile electrons can be induced by a gate voltage *V*_g_ under the narrow areas where the metal has no direct contact to the InSb surface. Thus, the wires and dots can be charged without direct contacts to the inversion-layer electrons since the InSb substrate has a finite resistivity, even at liquid helium temperatures. Holographic lithography can be used to fabricate wires and dots in arrays on macroscopic areas so that sufficiently high optical absorbance signals can be attained. A simple *k* · *p* approach to the concept of effective mass in low-dimensional systems leads to the following: (1) In a 2-D system, the electron mass depends on subband index and on momentum parallel to the layer; (2) in a 1-D system, the mass is only defined in the direction along the wire; (3) in a 0-D system, it is no longer meaningful. For 3-D electrons in a NGS, the mass increases with momentum away from the Γ-point, giving rise to nonparabolic effects. Merkt used an optically-pumped FIR laser to perform CR experiments. The average number of electrons in a dot and the mobility could be determined. The number of electrons per dot was found to be 3 ± 1 at low *V*_g_. The size of the dots is of the same order as the effective Bohr radius, 64 nm. Quantum dots have similarities with shallow donors in semiconductors; but unlike them, dots can have their size and electron number tuned by technological means. Quantization energies of up to 10 meV at wire widths below 100 nm were found by FIR spectroscopy.

*O. A. Pankrotov* (P. N. Lebedev Physical Institute) reviewed the concept of supersymmetry as applied to the electronic properties of band-inverted heterojunctions. Heterojunctions between semiconductors with mutually inverted bands contain massless spin-nondegenerate interface electron states. The universality of these states is due to the specific symmetry (the supersymmetry) of the effective Hamiltonian. Predictions of giant Landau splittings of the interface states and selection rules for optical transitions in a magnetic field were given.

*Paul Kruse* (Honeywell Sensors and Systems Development) gave a critical review of the physics and applications of high-*T*_c_ superconductors (HTSCs) for IR detection. With the discovery of HTSCs came the hope that they could be exploited to provide performance advantages over conventional IR detectors based upon semiconductors. Very long-wavelength photon detection at operating temperatures near that of liquid nitrogen was thought possible because the forbidden energy gap of HTSCs was much larger than that in metallic superconductors. Kruse reviewed three ways of estimating the energy gap in YBaCuO: from the Bardeen-Cooper-Schrieffer (BCS) theory, from optical measurements, and from electrical tunneling measurements. For a transition temperature of 90 K, the BCS theory predicts a gap of 27 meV. Optical measurements gave a range of values from 15.5 to 44 meV. However, it is evidently not possible to determine the gap from optical measurements due to residual absorption in the samples. The tunneling measurements also give inconsistent results. Three types of IR detectors based upon HTSC’s were presented: the transition-edge microbolometer, the non-equilibrium photo-effect, and photon-assisted tunneling detectors. Practical exploitation of these photon effects will be difficult and time consuming. However, success is likely, and the result will be a new class of very long-wavelength IR detectors and focal-plane arrays.

*Jacek Kossut* (Polish Academy of Sciences) reviewed the field of donor-electron correlations in DMSs with substitutional iron. Results for HgSe:Fe, Hg_1−_*_x_*Mn*_x_*Se:Fe, and HgSe_1−_*_x_*Te*_x_*:Fe were presented. Compounds involving HgSe substitutionally doped with Fe have interesting properties related to the unusual energy position of *d*-derived Fe states: the Fe^+2/+3^ state is superimposed on the conduction-band continuum of states creating a resonant donor state. It is energetically possible for the Fe impurity to donate one of its six *d*-electrons to the conduction band provided that there are unoccupied states available in the conduction band below the Fe level, *E*_Fe_. At low levels of Fe doping, the concentration *n* of free electrons in the conduction band is roughly proportional to the number of impurities, *N*_Fe_, while n becomes nearly constant above a certain critical concentration when the Fermi level *E*_F_ becomes “pinned” to the Fe resonant level *E*_Fe_. Anomalously high values of the electron mobility in this high-concentration region have been explained by the onset of spatial correlations of the donor charges, since the Coulomb repulsion between the ionized donors tends to keep them apart. Note that this correlation involves transfer of the quasi-localized electrons associated with the Fe impurities, not motion of the Fe atoms themselves. The existence of this correlation results in a dramatic reduction of the rate of scattering by ionized impurity potentials. The degree of suppression of the scattering rate is correctly reproduced by theoretical calculations that include these correlations. Other semiconducting materials (because of the resonant nature of their impurity states) may also exhibit a similar enhancement of their mobilities, e.g., DX centers associated with substitutional donors in GaAs and Cr in PbTe.

*Robert S. Allgaier* (formerly with the Naval Surface Warfare Center) presented an historical review covering research on a number of NGSs and semimetals during the 1945–1965 period, including IV–VI compounds (PbS, PbSe, PbTe, GeTe, and SnTe), Column-V semimetals (Sb and Bi), Bi_2_Te_3_, Te, Mg_2_Sn, graphite, *α*-Sn, InSb, and Hg_1−_*_x_*Cd*_x_*Te. The reference list is arranged chronologically and identifies the subject of each entry so that it can serve as an historical summary. The list includes many books, review articles, and conferences, plus selected individual references. Dr. Allgaier argued that the semiconductor era may have begun in 1931, when A. H. Wilson’s classic papers were published. Pre-war semiconductor research focused on oxides and other large-gap materials. Only in 1944 did the semiconductor category first appear as a subject classification in Physics Abstracts. The first III–V compound semiconductor paper appeared in 1952. Other events mentioned in the review of early NGS history include (1) superconductivity in GeTe and SnTe (first observed in 1964 and 1965); (2) the mid-1950s discovery that Bi_2_Te_3_ was an excellent material for thermoelectric generators and coolers; (3) the use of Te in the crystal receivers of the first two decades of this century; and (4) the early and rapid growth of InSb into the most popular member of the III–V family (it was relatively easy to grow and was quickly found to exhibit a number of very interesting properties).

Concluding remarks given by *Tony Stradling* (Imperial College) emphasized the new developments and trends occurring in the physics and technology of NGSs. He reviewed trends, selected highlights of the conference and predicted future efforts in the field. To detect trends, he analyzed the subject matter of the papers presented at this conference and at the two previous conferences in 1977 and 1981. It was quite apparent that technology subjects are now much more important. This is due to: (1) the burgeoning interest in narrow-gap detectors and sources and (2) the increasing dependence of both the physics and technology on sophisticated growth techniques such as MBE. The topic that pointed most obviously to the future was the Hamburg work on quantum dots. This work on InSb is in advance of that on GaAs/AlGaAs because of the low mass and high confinement energies involved. The remarkable and revolutionary political changes sweeping the world could reduce interest in military devices operating in the 10-µm wavelength range. However, high technology will still be important as squadrons and fleets are cut back. IR technology will be increasingly in demand in a wide variety of areas such as telecommunications, environmental and pollution monitoring, space-born astronomy, and in the field of medical diagnostics.

## 4. Conclusions

Narrow-gap semiconductors are important technological materials for infrared detector and laserdiode applications. The International Conference on Narrow-Gap Semiconductors and Related Materials, held in Gaithersburg, MD, June 12–15, 1989, served as an important vehicle for summarizing and disseminating up-to-date scientific research on these materials. Artificially structured materials hold great potential for new types of devices, as well as new materials for fundamental research.

## Figures and Tables

**Table 1:** Some representative narrow-gap semiconductors

Elements		Compounds				Alloys
	II–V	II–VI	III–V	IV–VI	V–VI	
Tellurium	BaAs_3_	HgS	InSb	PbS	Bi_2_Se_3_	Hg_1−_*_x_*Cd*_x_*Se
Selenium	CaAs_3_	HgSe	InAs	PbSe	Bi_2_Te_3_	Hg_1−_*_x_*Cd*_x_*Te
Gray Tin	Cd_3_As_2_	HgTe		PbTe	Sb_2_Se_3_	Hg_1−_*_x−y_*CD*_x_*Mny*_y_*Te
	Cd_3_P_2_			SnTe	Sb_2_Te_3_	Hg_1−_*_x_*Fe*_x_*Se
	*α*- and *β*-EuP_3_			GeTe		Hg_1−_*_x_*Fe*_x_*Te
	Zn_3_As_2_					Hg_1−_*_x_*Mn*_x_*Se
	Zn_3_P_2_					Hg_1−_*_x_*Mn*_x_*Te
						HgS*_x_*Se_1−_*_x_*
						Pb_1−_*_x_*Mn*_x_*Te
II–IV–V	II–IV					PbS_1−_*_x_*Se*_x_*
CdSnAs_2_	Mg_2_Sn					Pb_1−_*_x_*Sn*_x_*Se
						Pb_1−_*_x_*Sn*_x_*Te
Superlattices and Quantum Wells						PbSe*_x_*Te_1−_*_x_*
(Many combinations from the above lists, such as HgTe/CdTe, InAs/In_1−_*_x_*Ga*_x_*Sb, PbTe/Pb_1−_*_x_*Mn*_x_*Te, etc.)						Cd_3−_*_x_*Zn*_x_*As_2_
						(Cd_1−_*_x_*Mn*_x_*)_3_As_2_
						Pb_1−_*_x_*Ge*_x_*Te
						InAs*_x_*Sb_1−_*_x_*
						Pb_1−_*_x_*Cd*_x_*S
						Pb_1−_*_x−y_*Eu*_x_*S*_y_*Se
						Pb_1−_*_x−y_*Eu*_x_Te_y_*Se
						Pb_1−_*_x−y_*Cd*_x_*S*_y_*Se
						Pb_1−_*_x_*Eu*_x_*Te
						Pb_1−_*_x_*Eu*_x_*Se
						Pb_1−_*_x_*Sr*_x_*Se

**Table 2:** Previous conferences on narrow-gap semiconductors

Date	Place	Sponsors and financial support	Publication
Jan. 21, 1964	Columbia Univ., New York	Topical Conference APS	Proc. of Conf. on The Physics of Semimetals published in IBM J. Res. Dev., Vol. 8, 1964. One paper on IV–VI Compounds.
April 2–4, 1968	Univ. of Durham, England		Short report of Semimetals and Narrow-Gap Semiconductors Conference given by G. A. Saunders, in J. Phys., Colloque C4, Supp. to #11–12, Vol. 29, pp. 3–8, 1968.
July 15–18, 1968	Centre National de la Recherche Scientifique, Paris, France	C.N.R.S.	International Colloquium on IV–VI Compounds, J. Phys., Colloque C4, Supp. to #11–12, Vol. 29, 1968.
Mar. 20–21, 1970	Dallas, TX, USA	Texas Instruments, ONR, LTV Research Center, Topical Conf. of APS	Physics of Semimetals and Narrow-Gap Semiconductors Conference proceedings published in J. Phys. Chem. Solid, Vol. 32, Suppl. 1, 1971, pp. 1–568.
Mar. 24–25, 1972	Univ. of Pennsylvania, Philadephia, PA	Moore School of EE, Lab. for Res. on Struct. of Mat., Topical Conf. of APS	Physics of IV–VI Compounds and Alloys (Gordon & Breach, London, 1974).
Sept. 10–14, 1973	Nice, France Cardiff, Wales	Royal Society, Plessey Co. Limited, C.N.R.S.	Int. Conf. on The Physics of Semimetals and Narrow-Gap Semiconductors. Proceedings unpublished.
Sept. 12–15, 1977	Institute of Physics, Polish Academy of Sciences, Warsaw, Poland	Recognized by the Int. Union of Pure and Applied Physics (IUPAP)	Conf. Proc. published as Phys. of Narrow Gap Semicond. (PWN-Polish Scientific Publishing, Warsaw, 1978), pp. 1–481
Sept. 3–15, 1979	Université des Sciences et Techniques du Languedoc, Nimes, France	Various institutions and companies including European Research Office, IBM, ONR, Thomson CSF	Int. Summer School Proc. published as Narrow-Gap Semiconductors Physics and Applications, Lecture Notes in Physics, Vol. 133 (Springer-Verlag, New York, 1980), pp. 1–572.
Sept. 14–17, 1981	Johannes Kepler Univ., Linz, Austria	IUPAP, European Phys. Soc. Austrian Phys. Soc., IBM, Siemens, European Research Office, Austrian Fed. Ministry of Science and Research	Conf. Proc. published as Physics of Narrow-Gap Semiconductors, Lecture Notes in Physics, Vol. 152 (Springer-Verlag, New York, 1982), pp. 1–485.
